# The impact of patient age on the pharmacist’s recommendation of OTC anti-common cold products before and during the COVID-19 pandemic

**DOI:** 10.3389/fphar.2026.1856389

**Published:** 2026-08-11

**Authors:** Paulina Natalia Kopa-Stojak, Malgorzata Pietrusiewicz, Rafal Pawliczak

**Affiliations:** 1 Department of Immunopathology, Faculty of Medicine, Medical University of Lodz, Lodz, Poland; 2 Department of Apothecary Pharmacy, Faculty of Pharmacy, Medical University of Lodz, Lodz, Poland

**Keywords:** common cold, counseling, lung diseases, pharmaceutical preparations, pharmacy

## Abstract

**Introduction:**

A pharmacist is the first point of contact when a patient seeks the most effective over-the-counter treatment, usually for a self-diagnosed disease, especially in the case of difficult or limited access to medical practitioners. The aim of this study is to determine the impact of the patient’s age on the pharmacist’s recommendation of over-the-counter anti-common cold treatment before and during the COVID-19 pandemic.

**Materials and Methods:**

This study is based on a self-developed questionnaire about pharmacists’ recommendations for patients with cold symptoms collected in four large network community pharmacies in Lodz, Poland, before and during the COVID-19 pandemic. Pearson’s chi-square test and odds ratios with 95% confidence intervals were used to determine the relationship between the patient’s age and frequency of medication recommendations. The Mann–Whitney U test was used to determine differences in recommendations between the analyzed periods.

**Results:**

We report higher odds of pharmacists’ recommendations (odds ratio > 1; *p* < 0.01667) for ibuprofen with pseudoephedrine for patients aged 18–40 years, paracetamol with ascorbic acid, and phenylephrine HCl, acetylcysteine, other soluble products, and other tablets/capsules for patients aged 61–90 years before the COVID-19 pandemic. Moreover, there were higher odds of recommendations for paracetamol with ascorbic acid and phenylephrine HCl, inosines, and other soluble products for the 41–60 age cohort, and for homeopathic and herbal products for the 61–90 age cohort during the pandemic.

**Conclusion:**

Patients, especially older people who take several medications, require special attention when being advised about over-the-counter (OTC) medications. Our findings clearly demonstrated the constant need for improving pharmacists’ counseling regarding OTC medicines (i.e., age-, comorbidities-, and drug–drug-interaction-adapting recommendations) by training and increasing professional skills.

## 1 Introduction

The over-the-counter (OTC) medication market reached 125.3 billion USD in 2023 globally (Growth Market Reports, 2024), 137.4 billion USD in 2024, with estimations of approximately a 3.49% annual increase, to a projected 163.1 billion USD in 2029. Due to the seasonal increase in the incidence of common colds and coughs among the population, this sector is expected to dominate the OTC market (it was 25% of the OTC market in 2021). In 2022, the OTC cold and cough treatment sector reached 16.3 billion UDS with estimated growth at a CAGR of 6.1%–2030. In addition, the OTC analgesics sector reached 14.9 billion USD in 2022 with an expected increase at a CAGR of 5.7% from 2022 to 2030 (Mordor Intelligence, 2022).

Self-medication with OTC products is becoming increasingly common worldwide, with the prevalence ranging from 16.1% to 90.0% depending on the country and age group (Balbuena et al., 2009; Kasulkar and Gupta, 2015; Arrais et al., 2016; Håkonsen et al., 2016; Gama and Secoli, 2017; Abdi et al., 2018; Kassie et al., 2018; Lei et al., 2018). The main reasons for self-medication with non-prescription medicines were mild symptoms of disease (45% of patients), no effort to see a doctor (23% of patients), no time to see a doctor (12% of patients), and high costs of medical services (15% of patients) (Lei et al., 2018). Another study showed that easy access to pharmacy and mild illness symptoms were the most important reasons for self-medication (Chautrakarn et al., 2021). A logistic regression model showed that the factors of OTC use included female gender, multimorbidity, good, moderate, and poor self-reported health status, age (60–69 and 70–79 years old), and previous use of Rx treatment (Barrenberg et al., 2018). The main problems associated with self-medication with OTC products are overdosing, abuse, adverse drug reactions, antimicrobial resistance, and possible drug interactions, which may contribute to side effects and increased possibility of complications (Cooper, 2013; Watkins and Bonomo, 2016; Lasota et al., 2022).

A pharmacist is the first point of contact for patients seeking effective over-the-counter treatment, usually for a self-diagnosed disease, especially in the case of difficult or limited access to medical practitioners (Villako et al., 2012; Raal et al., 2013). The qualifications of pharmacists with regard to patient counseling differ between countries and depend on pharmacists’ training and local law (Montgomery et al., 2010; Iza Aptekarska, 2012; Mináriková et al., 2019). Based on qualifications, knowledge, experience, and skills, pharmacists may recommend available OTC products, their composition and dosage, and provide information about drug substitutes if a product is unavailable. In addition, a pharmacist may indicate an immediate need to visit a doctor, check the availability of drugs in other pharmacies, or recommend a different, more efficient drug than the one the patient initially asked for (Assemi et al., 2002; Waszyk-Nowaczyk and Simon, 2009; Kokane and Avhad, 2016).

With age, the risk of multiple chronic diseases (e.g., dyslipidemia, hypertension, cardiovascular disease, diabetes) increases, which results in the need to use multiple drugs concurrently (Vrdoljak and Borovac, 2015; Rodrigues and de Oliveira, 2016). Using many drug combinations may lead to increased risk of adverse drug reactions (ADRs) and drug–drug interactions (DDIs) (Kim et al., 2018; Martin et al., 2022). Moreover, the natural aging process leads to many physiological changes in the body (i.e., reduction of gastric motility, body mass and body water, and blood flow) and impairment of organ functions (i.e., kidney and liver function) that affect pharmacokinetics (absorption, distribution, metabolism and excretion) and pharmacodynamics (greater sensitivity to several drug classes but also adverse side effects (ASEs) to usual doses) of drugs (Drenth-van Maanen et al., 2020; Barry and Hughes, 2021). Therefore, due to age-related physiological changes, higher prevalence of chronic diseases and higher use of prescription and non-prescription medicines, older adults are particularly vulnerable to adverse health outcomes (including a 2–7 times higher prevalence of ADRs than younger adults) (Albert et al., 2014; Lehnbom et al., 2023). When choosing OTC drugs, it is important during pharmaceutical counseling to take into account the complexity of polypharmacy, increased risk of drug–drug and drug–disease interactions, and changes in pharmacokinetics and pharmacodynamics in the case of older customers (McKearney and Coleman, 2020).

The COVID-19 pandemic caused a significant increase in the sales of OTC medicines (Yáñez et al., 2021; Al Meslamani and Abdel-Qader, 2023). Patients, fearing SARS-CoV-2 infection and having limited access to health professionals, often chose OTC treatments, including antipyretics, vitamins, nose and throat hygiene products, and immunity-boosting products, for self-medication and preventive purposes (Karlsson et al., 2021; Romano et al., 2021). The *Petković et al.* study in Serbia showed that during the COVID-19 pandemic, 100% of respondents chose antipyretics, 99% chose analgesics, 82% chose decongestants, and 66% chose antitussives (Petković et al., 2022). Pharmacist counseling played a significant role during the COVID-19 outbreak, when access to medical practitioners was limited (Al-Quteimat and Amer, 2021). The COVID-19 pandemic also increased the demand for and consumption of medicines, and restrictions in global supply chains related to blockades and transport restrictions resulted in reduced availability of many OTC products. This situation also influenced changes in the pharmacists’ recommendations of OTC anti-common cold medicines (Ali and Alharbi, 2020; Barkur and Kamath, 2020; EMA, 2020).

Recent literature provides a limited number of studies on the use of OTC medicines by patients in different age groups (Kim et al., 2018; Sánchez-Sánchez et al., 2021; Lehnbom et al., 2023). Few studies compare the use of such OTC products before and during the COVID-19 pandemic (Yáñez et al., 2021; Kopa-Stojak et al., 2024). To our knowledge, no study combines an analysis of the impact of the COVID-19 pandemic on changes in OTC medicines recommendations considering patients’ age for these recommendations. Therefore, the aim of this study was to determine the impact of patients’ age on the pharmacists’ recommendation of over-the-counter anti-common cold treatment before and during the COVID-19 pandemic.

## 2 Materials and methods

### 2.1 Study design

This cross-sectional study was based on a self-developed questionnaire on pharmacist recommendations of over-the-counter anti-common cold treatment for patients with cold symptoms. According to Article 2(c) of Directive 2001/20/EC of the European Parliament and Council and the policies of the ethical committee of the Medical University of Lodz, this study was granted a formal exemption from ethical review because it was classified as a non-interventional observational trial, involved only minimal risk to participants, and did not collect sensitive or identifiable data. Implied consent to participate in the study was obtained through questionnaire completion.

### 2.2 Survey development and administration

This study is based on a short self-developed questionnaire with simple questions and multiple-choice options. The questionnaire collected minimal personal information (age and gender, which could potentially be classified as personally identifiable information (PII)). Privacy of the participants’ information was protected through all the stages of the study. The list of potentially recommended OTC products included in the questionnaire was based on a list of most frequently chosen OTC anti-common cold products in Poland. The list was created by pharmacists and medical practitioners who were a part of the research team. The study was conducted over the months with an increased number of common cold cases (December–February) to maximize the availability of patients with self-diagnosed common cold. Simultaneously with the questionnaire development, a guideline (short instruction) for pharmacists who would be involved in the counseling process was prepared. Before the launch, the study questionnaire was pilot-tested by the research team and by pharmacists on a small group of patients. The study was regularly supervised during regular meetings with the pharmacists (every 2–3 weeks), during which completed questionnaires were collected. To reduce the possibility of multiple participation in the study, the pharmacists were obliged to obtain information about previous involvement in similar surveys from patients during the counseling. The response rate was calculated after completion of the data collection.

The pharmacists who enrolled in this study underwent a course of training. During meetings with a member of the research team, they were familiarized with the questionnaire and guidelines. The pharmacists were also instructed how the consultation should be carried out and what information they should present to the study participants (including purpose of the study, target population, study time frame, contact information for survey administrator, handling of patients’ information, and protection of PII anonymity and confidentiality at each stage of the study), which should reduce the risk of recall bias.

### 2.3 Study population

The questionnaires were completed by a representative group of 27 pharmacists (chosen by random sampling) in four large network community pharmacies in Lodz, Poland, between December 2019 and February 2020 (Wave 1: before the COVID-19 pandemic). An outbreak of the COVID-19 pandemic in Poland in March 2020 (first cases) influenced the decision to collect the data during the pandemic period, between December 2021 and February 2022 (Wave 2: during the COVID-19 pandemic). The research team included pharmacists (n = 27 in both Wave 1 and Wave 2) who had been working in large network community pharmacies located both in Lodz and across Poland to allow generalization of the results.

The pharmacists completed the questionnaire for adult patients (aged ≥18 years) with self-diagnosed common cold (without consultation with medical practitioners) who had asked for advice on OTC anti-common cold treatment (no Rx or list of medicaments). Finally, the questionnaire was filled out only if the patient purchased the recommended OTC anti-common cold treatment. To obtain a representative sample of the study population, a systematic random sample method (every second patient asking for advice) was used. The training done before starting the study and regular supervision of pharmacists (especially by presentation of the survey and data expected to be collected, instruction on how to enter the data into the survey, regular collection of completed surveys, and checking the progress of work) helped to reduce the risk of social desirability bias (SDB) or recall bias and also to increase consistency in data collection among the pharmacists.

Based on the number of adult citizens of Lodz (≥18 years), 95% confidence interval Z score (1.96), margin of error (5%), and population proportion (50%; no previous similar studies in Lodz, Poland), a sample size of n = 384 was calculated (for Wave 1 and Wave 2, separately). In addition, the non-response rate/missing data was anticipated to be a maximum of 10% (Madley-Dowd et al., 2019); therefore, the final sample size was determined to be at least 427 patient questionnaires.

### 2.4 Study medications and variables

The first section of the data collection form gathered the data on age and sex (demographics) and the patient’s self-reported common cold symptoms (headache, temperature, cough, rhinitis, and/or fatigue). The second section of data collection form contained a list of possible recommended OTC products, divided into three categories (based on the formulation): (1) soluble OTC products (i.e., sachets, powders), (2) OTC products in form of tablets and capsules, and (3) add-on OTC products (i.e., sprays, gels, syrups, products for sore throat). The last section of the data collection form included the reasons for the recommendation of specific OTC products by pharmacists, which we discussed in our previous study (Kopa-Stojak et al., 2024). Supplementary Material S1 presents an English translation of the data collection form.

### 2.5 Statistical analysis

The proportion of patients in each age group who received each treatment was reported as a fraction of the number of cases to the total number of cases and the percentage of patients’ encounters with OTC anti-common cold recommendations. Pearson’s chi-square test was used to compare the frequency of receiving particular medications for patients in different age groups (18–40, 41–60, and 61–90). Odds ratios (OR) with 95% confidence intervals (CI) were calculated to determine the relationship between the patients’ age and the frequency of medication recommendations. In order to reduce the risk of type 1 error, Bonferroni correction was used in the analysis of the correlation between the patient’s age and OTC anti-common cold medication recommendation frequency. Results with a correlation coefficient ≥0.3 were considered clinically relevant. The Mann–Whitney U test was used to determine statistically significant differences between anti-common cold OTC recommendations before (December 2019–February 2020) and during (December 2021–February 2022) the COVID-19 pandemic. Statistical significance was set at p-value <0.05 and p-value <0.01667 (Bonferroni correction for subgroup analysis). Statistical analysis was performed using SigmaPlot 12.3 (Systat Software Inc., Palo Alto, CA, United States of America).

## 3 Results

### 3.1 Patient characteristics

The study was based on 1,000 pharmacist counselings of patients with common cold symptoms who sought advice on OTC treatment, including 502 sessions conducted before the COVID-19 pandemic (December 2019–February 2020) and 498 conducted during the COVID-19 pandemic (December 2021–February 2022). Most patients (58.1%) were women. Three of five patients were aged 18–40 years, and about 1/3 were aged 41–60 years. More than 2/3 (68.3%) of patients reported headache as their main common cold symptom. Almost 1/2 (49%) of them reported rhinorrhea, and more than 2/5 (43.4%) reported fatigue. One in three (35.6%) patients had cough, and almost 1/4 (23.2%) had a high temperature (Table 1).

**TABLE 1:** Demographics data.

Number of patients [n (%)]			
​	Before the COVID-19 pandemic [December 2019–February 2020]	During the COVID-19 pandemic [December 2021–February 2022	Total
Age	​	​	​
18–40	325 (64.7%)	281 (56.4%)	606 (60.6%)
41–60	146 (29.1%)	194 (40.0%)	340 (34.0%)
61–90	31 (6.2%)	23 (3.6%)	54 (5.4%)
Sex	​	​	​
Male	238 (47.4%)	181 (36.3%)	419 (41.9%)
Female	264 (52.6%)	317 (63.7%)	581 (58.1%)
Reported common cold symptoms	​	​	​
Cough	177 (35.3%)	179 (35.9%)	356 (35.6%)
Fatigue	165 (32.9%)	269 (54.0%)	434 (43.4%)
Headache	389 (77.5%)	294 (59.0%)	683 (68.3%)
Rhinorrhea	308 (61.4%)	182 (36.5%)	490 (49.0%)
Temperature	140 (27.9%)	92 (18.4%)	232 (23.2%)

### 3.2 Most recommended OTC anti-common cold medications based on patient age before the COVID-19 pandemic

#### 3.2.1 Recommendations for patients aged 18–40 years

Most often, the pharmacists declared proposing products containing a combination of paracetamol with phenylephrine HCl and pheniramine maleate (in 39/325 (12%) cases), ascorbic acid and pheniramine maleate (in 35/325 (10.8%) cases), ascorbic acid and phenylephrine HCl (in 36/325 (11.1%) cases), phenylephrine HCl (in 37/325 (11.4%) cases), or phenylephrine HCl and guaifenesin (in 39/325 (12%) cases). For 119/325 (36.6%) patients in this group, the pharmacists also recommended medications containing inosine pranobex. In addition, 107/325 (32.9%) of the patients received products containing xylometazoline, 50/325 (15.4%) received ambroxol HCl, and 55/325 (16.9%) received tablets containing ibuprofen and pseudoephedrine or tablets with a combination of paracetamol with pseudoephedrine and dextromethorphan hydrobromide. In addition, 42/325 (12.9%) patients received products for sore throat (including antiviral/antibacterial agents and choline salicylate) (Table 2).

**TABLE 2:** Comparison of anti-common cold OTC treatment recommendations in different age groups before and during the COVID-19 pandemic.

OTC anti-common cold products		Recommendations December 2019–February 2020 [n]	Recommendations December 2021–February 2022 [n]	Δ%	p
Patients aged 18–40 years					
Soluble	p-mol/asc ac/phen mal	35	15	42.9	0.015
	p-mol/asc ac/phen HCl	36	16	44.4	0.018
	Acet sal ac/phen HCl	9	6	66.7	0.617
	Meta mg	17	5	29.4	0.024
	p-mol/phen HCL/phen mal	39	16	41.0	0.007
	p-mol/phen HCl	37	24	64.9	0.274
	p-mol/phen HCl/gua	39	9	23.1	<0.001
	Other	11	14	127.3	0.325
Tablets/capsules	p-ephed/tiopro	7	8	114.3	0.585
	Acet sal ac	13	9	69.2	0.602
	Acet sal ac/asc ac	14	16	114.3	0.433
	Acet sal ac/p-ephed	8	2	25	0.092
	p-mol/phen HCl/asc ac	14	11	78.6	0.809
	Homeopathic product	28	6	21.4	<0.001
	Inosine pranobex	119	28	23.5	<0.001
	p-mol/p-ephed/dex HBr	55	20	36.4	<0.001
	Herbal product	9	5	55.6	0.419
	Ibuprofen/p-ephed	55	39	70.9	0.303
	p-mol/p-ephed/chlorphen	13	7	50	0.109
	Other	10	64	640	<0.001
Add-on	Acetylcysteine	7	12	171.4	0.136
	Fexofenadine HCl	18	10	55.6	0.248
	Cetirizine di-HCl	7	6	85.7	0.988
	Antibacterial/antifungal agent	42	2	4.8	<0.001
	Choline salicylate	12	2	16.7	0.015
	Ambroxol HCl	50	10	20	<0.001
	Loratadine	5	3	60	0.614
	Bromhexine HCl	23	42	182.6	0.002
	Xylometazoline HCl	107	57	53.3	<0.001
	Oxymetazoline HCl	24	1	4.2	<0.001
	Other	11	90	818.2	<0.001
Patients aged 41–60 years					
Soluble	p-mol/asc ac/phen mal	13	12	92.3	0.343
	p-mol/asc ac/phen HCl	20	27	135	0.955
	Acet sal ac/phen HCl	2	4	200	0.634
	Meta mg	7	7	0	0.587
	p-mol/phen HCl/phen mal	22	13	59.1	0.012
	p-mol/phen HCl	18	12	66.7	0.049
	p-mol/phen HCl/gua	24	4	16.7	<0.001
	Other	6	21	350	0.024
Tablets/capsules	p-ephed/tiopro	0	2	200	0.221
	Acet sal ac	3	10	333.3	0.141
	Acet sal ac/asc ac	3	9	300	0.202
	Acet sal ac/p-ephed	6	1	16.7	0.021
	p-mol/phen HCl/asc ac	11	8	72.7	0.176
	Homeopathic product	3	8	266.7	0.400
	Inosine pranobex	57	44	77.2	<0.001
	p-mol/p-ephed/dex HBr	22	15	68.2	0.021
	Herbal product	2	10	500	0.004
	Ibuprofen/p-ephed	13	22	169.2	0.343
	p-mol/p-ephed/chlorphen	7	9	128.6	0.948
	Other	7	59	842.9	<0.001
Add-on	Acetylcysteine	6	8	133.3	0.996
	Fexofenadine HCl	4	6	150	0.850
	Cetirizine di-HCl	2	7	350	0.205
	Antibacterial/antifungal agent	17	8	47.1	0.009
	Choline salicylate	5	3	60	0.260
	Ambroxol HCl	19	16	84.2	0.113
	Loratadine	1	2	200	0.739
	Bromhexine HCl	13	30	230.8	0.072
	Xylometazoline HCl	44	35	79.5	0.007
	Oxymetazoline HCl	10	1	10	0.001
	Other	3	76	2,533.3	<0.001
Patients aged 61–90 years					
Soluble	p-mol/asc ac/phen mal	1	2	200	0.369
	p-mol/asc ac/phen HCl	9	3	33.3	0.266
	Acet sal ac/phen HCl	1	2	200	0.369
	Meta mg	3	2	66.7	0.928
	p-mol/phen HCl/phen mal	1	2	200	0.369
	p-mol/phen HCl	4	2	50	0.651
	p-mol/phen HCl/gua	4	3	75	0.988
	Other	8	4	50	0.485
Tablets/capsules	p-ephed/tiopro	0	0	0	1.0
	Acet sal ac	1	1	0	0.334
	Acet sal ac/asc ac	0	2	200	0.101
	Acet sal ac/p-ephed	0	1	100	1.0
	p-mol/phen HCl/asc ac	4	2	50	0.928
	Homeopathic product	4	4	0	0.646
	Inosine pranobex	6	4	66.7	0.881
	p-mol/p-ephed/dex HBr	3	0	300	0.136
	Herbal product	0	3	300	0.041
	ibuprofen/p-ephed	4	2	50	0.651
	p-mol/p-ephed/chlorphen	3	0	300	0.136
	Other	5	7	140	0.209
Add-on	Acetylcysteine	8	0	800	0.017
	Fexofenadine HCl	3	0	300	0.232
	Cetirizine di-HCl	1	0	100	0.414
	Antibacterial/antifungal agent	8	2	25	0.119
	Choline salicylate	2	1	50	0.846
	Ambroxol HCl	6	2	33.3	0.293
	Loratadine	0	1	100	0.259
	Bromhexine HCl	1	3	300	0.179
	Xylometazoline HCl	7	5	71.4	0.970
	Oxymetazoline HCl	2	0	200	0.232
	Other	4	8	200	0.055

Abbreviations: *acet sal ac*, acetylsalicylic acid; *asc ac*, ascorbic acid; *chlorphen*, chlorpheniramine; dex *HBr*, dextromethorphan hydrobromide; *gua*, guaifenesin; *meta mg*, metamizole magnesium; *p-mol*, paracetamol; *phen HCl*, phenylephrine HCl; *phen mal*, pheniramine maleate; *p-ephed*, pseudoephedrine; *tripro*, triprolidine.

#### 3.2.2 Recommendations for patients aged 41–60 years

In 57/146 (39%) cases, the patients received products containing inosine pranobex, and 22/146 (15.1%) took products containing paracetamol with pseudoephedrine and dextromethorphan hydrobromide. In addition, for more than half of those patients, the pharmacists proposed drugs containing a combination of paracetamol with phenylephrine HCl and guaifenesin (recommended in 24/146 (16.4%) cases), phenylephrine hydrochloride and pheniramine maleate (recommended in 22/146 (15.1%) cases), phenylephrine HCl (recommended in 18/143 (12.4%) cases), or ascorbic acid and phenylephrine HCl (recommended in 20/146 (13.7%) cases). Finally, medications with xylometazoline were received by 44/146 (30.1%) patients. In 19/146 (13%) cases, the pharmacists recommended medications with ambroxol HCl (Table 2).

#### 3.2.3 Recommendations for patients aged 61–90 years

Interestingly, for 17/31 (54.8%) patients older than 60 years, the pharmacists recommended drugs other than the most common anti-cold medications listed in the questionnaire for each group of products. For this group of patients, the most common choice were products containing acetylcysteine (recommended in 8/31 (25.8%) cases), xylometazoline HCl (advised in 7/31 (22.6%) cases), ambroxol HCl (in 6/31 (19.4%) cases), inosines (recommended in 6/31 (19.4%) cases), and products for sore throat treatment (recommended in 8/31 (25.8%) cases). Moreover, 13/31 (41.9%) patients older than 60 years received products containing a combination of paracetamol with phenylephrine HCl and ascorbic acid (for both soluble and tablets/capsules formulation). Paracetamol with phenylephrine HCl and guaifenesin was advised in 4/31 (12.9%) cases. Finally, in 4/31 (12.9%) cases, paracetamol and phenylephrine HCl were recommended. Homeopathic remedies and ibuprofen with pseudoephedrine were each advised in 4/31 (12.9%) patients (Table 2).

### 3.3 Changes in medications advised by the pharmacists based on the patient’s age during the COVID-19 pandemic

#### 3.3.1 Recommendations for patients aged 18–40 years

In a similar period during the COVID-19 pandemic, the pharmacists most often recommended drugs other than the most common anti-cold medications listed in the questionnaire for each group of products (in 168/281 (59.8%) cases). In addition, one in five (57/281 (20.3%) cases) patients received products containing xylometazoline HCl, and one in ten patients (28/281 (10%) cases) received medications with inosine pranobex. Finally, during the COVID-19 pandemic, the pharmacists often advised products containing ibuprofen and pseudoephedrine (in 39/281 (13.9%) cases) and bromhexine HCl (in 42/281 (14.9%) cases) (Table 2).

The Mann–Whitney U test showed a statistically significant increase in recommendation of other anti-common cold treatments in the form of tablets/capsules (*p* < 0.001), other add-on treatment (*p* < 0.001) and bromhexine HCl (*p* = 0.002) and significant reduction in recommendations of paracetamol-containing products (including paracetamol with: ascorbic acid and phenylephrine maleate (*p* = 0.015), ascorbic acid and phenylephrine HCl (*p* = 0.018), phenylephrine maleate and phenylephrine HCl (*p* = 0.007), phenylephrine HCl and guaifenesin (*p* < 0.001), pseudoephedrine and dextromethorphan HBr (*p* < 0.001), and pseudoephedrine and chlorpheniramine (*p* < 0.001)). There was a significant reduction in the recommendation frequency of metamizole magnesium (*p* = 0.024), inosines (*p* < 0.001), homeopathics (*p* < 0.001), choline salicylate (*p* = 0.015), xylometazoline HCl (*p* < 0.001), and oxymetazoline HCl (*p* < 0.001) compared with an analogous period before the COVID-19 pandemic (Table 2).

#### 3.3.2 Recommendations for patients aged 41–60 years

During the COVID-19 pandemic, for 156/194 (80.4%) patients, the pharmacists recommended drugs other than the most common anti-cold medications listed in the questionnaire for each group of products. In addition, for this group of patients, the most commonly recommended products contained inosine pranobex (in 44/194 (22.7%) cases), xylometazoline HCl (in 35/194 (18%) cases), and bromhexine HCl (in 30/194 (15.5%) cases). Finally, in 27/194 (13.9%) cases, the pharmacists recommended products containing a combination of paracetamol with ascorbic acid and phenylephrine HCl (Table 2).

The Mann–Whitney U test showed a statistically significant increase in recommendation of herbal products (*p* = 0.004), other soluble products (*p* = 0.024), other treatments in the form of tablets/capsules (*p* < 0.001) and other add-on products (*p* < 0.001). There was a significant reduction in recommendation frequency of products containing paracetamol (including the combination of paracetamol with ascorbic acid and phenylephrine maleate (*p* = 0.012), phenylephrine HCl (*p* = 0.049), phenylephrine HCl and guaifenesin (*p* < 0.001), pseudoephedrine and dextromethorphan HBr (*p* = 0.021)), acetylsalicylic acid with pseudoephedrine (*p* = 0.021), inosines (*p* < 0.001), antibacterial/antifungal agents (*p* = 0.009), xylometazoline HCl (*p* = 0.007), and oxymetazoline HCl (*p* = 0.001) (Table 2).

#### 3.3.3 Recommendations for patients aged 61–90 years

For patients older than 60 years, similarly to the analogous period before the pandemic, the pharmacists most often advised drugs other than the most common anti-cold medications listed in the questionnaire for each group of products (in 19/23 (82.6%) cases). Moreover, almost one in three patients received recommendations for homeopathic and herbal products (in 7/23 (30.4%) cases). For one in five patients, the pharmacists recommended products containing xylometazoline HCl (in 5/23 (21.7%) cases). Products with inosine pranobex were advised in 4/23 (17.4%) cases. Finally, for more than a third of these patients, the pharmacists proposed drugs containing a combination of paracetamol with phenylephrine HCl and guaifenesin (recommended in 3/23 (13%) cases), ascorbic acid and phenylephrine HCl (recommended in 3/23 (13%) cases), or bromhexine HCl (advised in 3/23 (13%) cases). The Mann–Whitney U test showed a statistically significant increase in recommendation of herbal products (*p* = 0.041) and a reduction in recommendation of acetylcysteine (*p* = 0.017) (Table 2).

### 3.4 Correlation of patient’s age and OTC anti-common cold medications recommendation frequency before the COVID-19 pandemic

**FIGURE 1:**
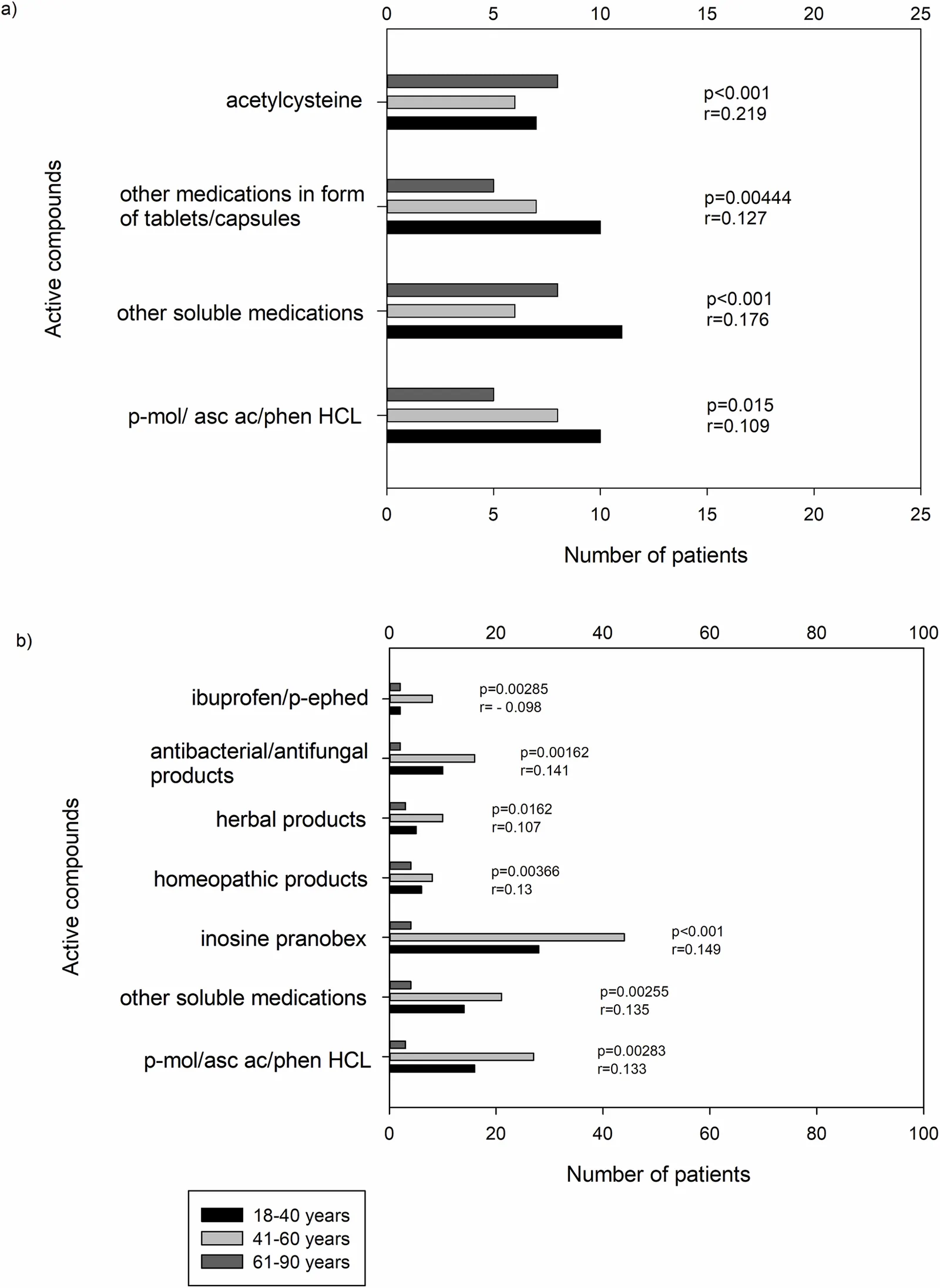
Anti-common cold OTC treatment correlated with patient age. A significant correlation between patient age and the frequency of recommending anti-common cold products in the group of **(a)** 502 patients before the COVID-19 pandemic and **(b)** 498 patients during the COVID-19 pandemic, who asked the pharmacists for advice on OTC medications. The results are presented as raw numbers of patients who used anti-common cold medications. The Pearson chi-square test was used to compare the frequency of receiving particular medications by patients of different ages. Data with p-values <0.01667 (Bonferroni correction) were considered statistically significant. Abbreviations: asc ac, ascorbic acid; p-mol, paracetamol; phen HCl, phenylephrine HCl.

The Pearson chi-squared test showed a negligible positive correlation between the patient’s age and frequency of prescribing anti-common cold products containing a combination of soluble paracetamol with ascorbic acid and phenylephrine HCl (r = 0.109, *p* = 0.015) for the soluble form (for patients 61–90 years old OR = 3.0317, 95% CI = 1.3296–6.9124, *p* = 0.0084). We observed similar results for other soluble products (r = 0.176; *p* < 0.001 (for patients 61–90 years old OR = 9.8913, 95% CI = 3.8390–25.4854, *p* < 0.001)), other products in form of tablets/capsules (r = 0.127; *p* = 0.00444 (for patients 61–90 years old OR = 5.1357, 95% CI = 1.7570–15.0119, *p* = 0.0028)), and acetylcysteine (r = 0.219, *p* < 0.001 (for patients 61–90 years old OR = 12.2542, 95% CI = 4.6210–32.4962, *p* < 0.001)). Finally, there was a statistically significant increase in recommendation frequency of other add-on treatments (OR = 4.8360, 95% CI = 1.4901–15.6920, *p* = 0.0082) for patients 61–90 years old (Figure 1a; Supplementary Table S1).

### 3.5 Correlation of patient’s age and OTC anti-common cold medications recommendation frequency during the COVID-19 pandemic

The Pearson chi-squared test showed that there was a negligible positive correlation between the patient’s age and frequency of prescribing anti-common cold products containing a combination of soluble paracetamol with ascorbic acid and phenylephrine HCl (r = 0.133; p = 0.00283 (for patients 41–60 years old OR = 2.4422, 95% CI = 1.3175–4.5268, *p* = 0.0046)) and other soluble OTC anti-cold products (r = 0.135; *p* = 0.00255). We noted similar results for products containing inosines (r = 0.149; *p* < 0.001 (for patients 41–60 years old OR = 2.5117, 95% CI = 1.5280–4.1286, *p* = 0.0003)), homeopathic (r = 0.13; *p* = 0.00366 (for patients 61–90 years old OR = 6.9624, 95% CI = 2.0924–23.1669, *p* = 0.0016)), and herbal (r = 0.107; *p* = 0.0162) medications. Finally, there was a negligible significant positive correlation between the patient’s age and frequency of prescribing anti-cold products containing antibacterial/antifungal agents (r = 0.141; p = 0.00162) and a negligible negative correlation between the patient’s age and frequency of prescribing ibuprofen and pseudoephedrine (r = − 0.098; *p* = 0.00285). In addition, there was a statistically significant increase in recommendation frequency of paracetamol with phenylephrine HCl and guaifenesin (OR = 5.3538, 95% CI = 1.4120–20.3002, *p* = 0.0136) for patients 61–90 years old (Figure 1b; Supplementary Table S2).

## 4 Discussion

Our study demonstrated some differences in pharmacists’ recommendations of OTC anti-common cold medicines among patients of different ages. Before the COVID-19 pandemic, the pharmacists mostly recommended paracetamol-containing products (80%–90%) for all patients, and alpha-mimetics (approximately 40%), inosine pranobex (approximately 37%), and mucolytics (approximately 26%) for patients aged 18–40 and 41–60 years. In addition, there was a significant increase in mucolytics (almost 50%), sore throat products (approximately 32%), and other soluble anti-common cold treatments (almost 26%), and a reduction in alpha-mimetics (29%) recommendations for patients aged 61–90 years. Our results showed that there were higher odds of pharmacists’ recommendations (OR>1; *p* < 0.01667) for ibuprofen with pseudoephedrine for patients aged 18–40 years, paracetamol with ascorbic acid, phenylephrine HCl, acetylcysteine, other soluble products, and other products in the form of tablets/capsules in the 61–90 age cohort.

Paracetamol remains a recommended analgesic, especially for older adults and frail patients (Freo et al., 2021). In the case of most anti-common cold products, it is combined with other active compounds, such as guaifenesin, chlorpheniramine, and phenylephrine, to treat the most common cold symptoms. However, such active compounds may be associated with many side effects (Albrecht et al., 2017; Kiran et al., 2018). In addition, long-term use of paracetamol may increase the risk of liver failure as well as cardiovascular, gastrointestinal, and renal adverse events (Freo et al., 2021). Risk factors of toxicity of paracetamol include older age, frailty, weight ≤50 kg, malnourishment, and renal or liver impairment (Yap et al., 2023). However, preferential pricing compared to drugs for common cold symptoms sold separately (with higher efficiency) may be a factor in the high sales of paracetamol-containing products (Salisbury-Afshar, 2012). Non-steroidal anti-inflammatory drugs (NSAIDs), such as ibuprofen, have proven better effectiveness against sneezing and fever than paracetamol (Kim et al., 2015). However, short-term use of NSAIDs may increase mild gastrointestinal adverse events, and long-term use may cause gastrointestinal ulceration or bleeding, which limits their application for people with peptic ulcer disease or prone to gastrointestinal bleeding (Monteiro et al., 2022).

There was no reasonable recommendation of mucolytics for upper respiratory viral infection (Unuvar et al., 2010) without analyzing cough characteristics (Irwin et al., 2006; Malesker et al., 2017). Acetylcysteine possesses mucolytic properties, but also antioxidant, anti-inflammatory, and immune-modulating characteristics (Shi and Puyo, 2020). Thus, it may be useful as an adjuvant in treating many medical conditions, including influenza or the common cold (Šalamon et al., 2019; Schwalfenberg, 2021). However, its usage should be avoided in cases of stomach ulcers or bleeding, high blood pressure, a low-salt diet, congestive heart failure, and kidney disease (Acetylcysteine Uses, Side Effects & Warnings, 2023). The main symptoms of the common cold are often accompanied by a sore throat, which in most cases is associated with viral infection. Therefore, symptomatic treatment with sore throat products instead of antibiotic treatment is advisable (Klimek and Sperl, 2015; Coutinho et al., 2021). Despite existing findings that inosine pranobex (IP) provides immunomodulatory activity (enhancing activity of NK cells, proliferation of T-cell lymphocytes, and secretion of pro-inflammatory cytokines) and can inhibit growth of several viruses (Beran et al., 2016; Sliva et al., 2019), such an antiviral agent is not clinically effective in treating patients with common cold (Jefferson and Tyrrell, 2001).

During the COVID-19 pandemic, there was an increase in recommendation frequency of other tablets/capsules (advised in 25%–30% of patients) and add-on (advised in 35% of patients) OTC anti-common cold products for all patients, and acetylsalicylic acid (advised in 26% of patients) for patients aged 61–90 years. There was a similar recommendation frequency of mucolytics (advised in 24% and 29% of patients, respectively) for patients aged 18–40 and 41–60 years and alpha-mimetics (advised for 22% of patients) for patients aged 61–90 years. Finally, there was a reduction in recommendation frequency of paracetamol-containing products (advised in 42%, 52% and 61% of patients respectively) for all patients, alpha-mimetics (advised in 21% of patients) for patients aged 18–40 years, inosine pranobex (advised in 23% of patients) for patients aged 41–60, and mucolytics (advised in 26% of patients) for patients aged 61–90 years. In addition, during the COVID-19 pandemic, there were higher odds (OR > 1; *p* < 0.01667) for pharmacists to recommend paracetamol with ascorbic acid and phenylephrine HCl, inosines, other soluble products for the 41–60 age cohort, and homeopathic and herbal products for the patients aged 61–90.

At the beginning of the COVID-19 pandemic, increased demand and consumption of medicines and restrictions in the global supply chain (related to blockades and transport restrictions) resulted in reduced availability of many OTC products, such as antipyretics, antihistamines, or cough suppressants in pharmacies. This situation could be the reason for significant differences in pharmacists’ recommendations for OTC products between the analyzed periods (Ali and Alharbi, 2020; Barkur and Kamath, 2020; EMA, 2020). With the outbreak of the COVID-19 pandemic, several studies appeared regarding the potential use of inosine pranobex in patients in the early phase of Sars-CoV-2 infection. The therapeutic potential of IP was explained by its effect on the activation of NK cells, enhancement of the expression of the NKG2D ligand, and interaction with the surface adenosine A2A receptor and PPAR-ɣ activation. Therefore, increased interest in the potential use of inosine pranobex in fighting COVID-19 may have contributed to maintaining a higher level of its recommendations by pharmacists to patients in the 41–60 year cohort during the pandemic (Isakov, 2021; Jayanthi et al., 2022). However, using IP may lead to transient elevation of serum and urinary uric acid levels (as a result of the catabolism of the inosine component into uric acid), which may negatively affect patients with gout, nephrolithiasis, or renal impairment. In addition, IP increases the potential risk for fluid retention and electrolyte shifts in patients with severe cardiac conditions (Basile et al., 2022).

Current clinical trials have shown an unclear/possible weak beneficial effect, albeit with questionable clinical relevance, of using some natural products, that is, garlic, echinacea, or *Pelargonium sidoides* extract, to prevent or treat the common cold (Zhang et al., 2007; Timmer et al., 2013; Karsch-Völk et al., 2014; Lissiman et al., 2014). Due to possible drug–drug interactions, herbal medicines may pose a serious risk for patients who commonly use prescription medicines for hypertension or diabetes (de Souza Silva et al., 2014). The most common reported interactions among elderly patients were bleeding caused by a combination of ginkgo biloba, garlic, or ginseng with aspirin or warfarin (Agbabiaka et al., 2017) or increased bioavailability and intravenous clearance of midazolam by *Echinacea* (Agbabiaka et al., 2018). Despite findings that homeopathy increased the severity of complaints for most of the patients within the first months of the treatment (Teut et al., 2010), there are unclear/unlikely benefits of its use in common cold treatment (Allan and Arroll, 2014). Furthermore, the use of acetylsalicylic acid, especially for elderly patients, increases the risk of hemorrhagic stroke and extracranial bleeding (Saad et al., 2019).

Pharmaceutical counseling should allow the selection of appropriate over-the-counter medications tailored to the patient’s characteristics (i.e., age and medication history) and medicines taken (Royal Pharmaceutical Society, 2024). Each pharmacist is guided in their decisions by their knowledge, skills, and essential key factors and preferences regarding individual medicines (Abraham and Chmielinski, 2018; Gilson et al., 2019; Tong and Aslani, 2019). In the study by *Halila et al.*, almost all Brazilian pharmacists determined that drug efficiency (97%), followed by drug adverse reactions (62.3%) and previous experience with the products (54.8%), are the most important factors considered during the OTC medicine counseling. Almost half of the pharmacists (46%) indicated that product price plays a significant role when recommending OTC drugs. Every fifth pharmacist mentioned customer’s preferences (21.2%) and product profit (18.8%) as factors significantly related to their selection of OTC products (Halila et al., 2015). Among the respondents in the *Moritz et al.* study in Germany, the most important factor in practical OTC recommendation was individual patient situation (93%), followed by user-friendliness, patient wish, personal experience, and recommendations from guidelines (90%–64%). Almost half of the respondents mentioned clinical trial data (48%), colleagues’ experience (45%), and feedback from other patients (41%) as decisive factors when recommending OTC products. Finally, economic factors, that is, purchasing condition/price, sales representatives’ information, positioning behind the counter, or familiarity through advertising, were listed as least important (13%–27%) during the patient’s counseling (Moritz et al., 2019).

However, pharmaceutical consultation is not conducted or carried out sufficiently in every case. The study by *Negru et al.* showed that pharmacists’ level of counseling on OTC medicines did not exceed 34% and included mainly drug administration and dosage (34%), drug–food interactions (30.7%), precautions and contraindications (30.7%), presence of other current medical conditions, allergies and treatments (11.2%), and secondary effects or adverse reactions (10.8%) (Negru et al., 2012). In the study by *Abu-Zaid et al.*, the pharmacists indicated that the main reason for the lack of effective consultation is lack of time or too little time for each patient (33.6%) due to pharmacists’ work overload (32.8%). Every fourth pharmacist indicated lack of designated places for counseling (24%) and insufficient knowledge about diseases (23.2%). More than one-fifth of pharmacists cited insufficient knowledge about medications (21.6%) as a problem in adequate patient consultation (Abu-Zaid et al., 2021).

There is a critical need to improve pharmacists’ counseling about OTC medicines. A range of training opportunities may be used to support current practice on supplying OTC products, including education on the efficiency of OTC products based on clinical trials, utilization of a symptom-driven approach, a service-based system apart from a medication-centered system only, and supply of OTC medicines in polypharmacy and comorbidity conditions with a focus on possible ADRs and DDRs (Allen and Simenson, 2013; Mináriková et al., 2019; Tong and Aslani, 2019).

Our study has some limitations. First, the pharmaceutical consultation was not subject to any supervision (audio/video monitoring), nor was it checked with the help of a mystery shopper. For this reason, we are unable to confirm whether each patient who met the study inclusion criteria was consulted and the data questionnaire was completed. Moreover, self-reporting of the patients’ data by the pharmacists may have increased the risk of bias (i.e., social desirability bias or recall bias). However, training the enrolled pharmacists before starting the study and regular supervision of the work progression helped to reduce the probability of such risk and increased consistency in data collection among the pharmacists. Second, data on recommended medicines were collected only if the patient had asked for advice for themselves (and not for another person) and purchased the recommended products, which may increase the risk of selection bias. However, we could not confirm whether the products had been purchased in another pharmacy. Third, due to the large number of OTC anti-common cold products available in Poland, their number in the survey was reduced to the most frequently sold/recommended products. Additionally, for each group of drugs (soluble, tablets/capsules and add-on OTC products), an “other” option was added, but without any space to specify the name of the product/active substance. For this reason, we were unable to determine which products had been recommended, especially during the COVID-19 pandemic, when the number of recommendations for other soluble, tablets/capsules and add-on products increased significantly. The questionnaire did not contain data on comorbidities or other medications. Adding such data would enable the analysis of whether the pharmaceutical consultations were also adapted to individual patient characteristics, and not only based on age, effectiveness, or preferential price of individual OTC drugs. Finally, due to significant changes in the availability of medicines, including OTC products, data from the beginning of the COVID-19 pandemic (December 2021–February 2022) were not combined with data from the pre-pandemic period (December 2019–February 2020) but discussed in separate subsections.

## 5 Conclusion

Patients who take several medications at the same time, especially older people, require special attention when selecting OTC medications. The individual patient and medication factors determine the need, the type, and the amount of required pharmacist’s counseling. The pharmaceutical consultation does not always finalize in the purchase of an OTC anti-common cold medicine tailored to the patient’s age (i.e., high recommendation frequency of acetylsalicylic acid for patients aged 61–90 years) or its clinical effectiveness (i.e., high recommendation frequency of inosines, homeopathic, and herbal products). For this reason, continuous training for pharmacists (i.e., training focusing on the safety of OTC products in relation to the patient’s age, comorbidities, or drug–drug interactions; or the clinical effectiveness of medicines) and improvement of their professional skills is necessary to improve the quality of their counseling. There is a constant need for monitoring of OTC product recommendations, especially in cases such as the evolving COVID-19 pandemic.

## Data Availability

The original contributions presented in the study are included in the article/Supplementary Material, further inquiries can be directed to the corresponding author.
