# Supplementary material for: The impact of patient age on the pharmacist’s recommendation of OTC anti-common cold products before and during the COVID-19 pandemic
Source: Front Pharmacol. 2026 Aug 11;17:1856389. doi: 10.3389/fphar.2026.1856389 (PMC13503212; doi:10.3389/fphar.2026.1856389)
Supplement: Supplementary file 1 [file Supplementaryfile1.docx]

**QUESTIONNAIRE**

**AGE**

**□** 18-40	**□** 41-60	**□** 61-90

**SEX**

**□** women	**□** men

**SYMPTOMS**

**□** headache	**□** fever

**□** rhinorrhoea	**□** cough	**□** fatigue

**BASIC MEDICATIONS**

**Soluble formulation:**

**□** Apap Anti-Cold	**□** Coldrex Max Grip	**□** Febrisan

**□** Fervex	**□** Gripex Hot	**□** Gripex Hot Max

**□** Polopiryna Complex	**□** Pyralgina Sprint	**□**Theraflu Extra Grip

**□**Theraflu Sinus	**□**Vicks	**□**other

**Tablets/Capsules:**

**□** Acatar Anti Tabs	**□**Aspirin	**□** Aspirin C

**□** Aspirin Complex	**□** Coldrex Max Grip	**□** Eloprine

**□**Engystol	**□** Esberitox	**□** Gripex

**□** Gripex Max	**□** Groprinosin	**□**Ibuprom Sinus

**□** Neosine	**□** Oscillococcinum	**□** Tabcin

**□** others

**ADD-ON MEDICATIONS (different formulations)**

**□** ACC	**□** Allegra	**□** Allertec

**□** Chlorchinaldin	**□** Cholinex	**□** Deflegmin

**□** Flavamed	**□** Flegamina	**□** Loratan

**□** Mucosolvan	**□** Neo Angin	**□** Otrivin

**□** Strepsils	**□** Sudafed Xylo spray	**□** Vicks spray

**□** Xylorhin	**□** others

**DECISION RATIONALE**

**□**medicament price	**□** in-depth patient’s interview

**□** knowledge and belief about medicine effectiveness

**□** other (marketing policy, bonuses for sale achievements, etc.)
