# Supplementary material for: The impact of patient age on the pharmacist’s recommendation of OTC anti-common cold products before and during the COVID-19 pandemic
Source: Front Pharmacol. 2026 Aug 11;17:1856389. doi: 10.3389/fphar.2026.1856389 (PMC13503212; doi:10.3389/fphar.2026.1856389)
Supplement: Supplementary file 2 [file Table1.docx]

**Supplementary Table S1. Correlation of patient’s age and OTC anti-common cold medications recommendation frequency before COVID-19 pandemic.**

| OTC anti-common cold products | | Pearson chi^2^ | | Patients 18-40 years-old | | | | Patients 41-60 years-old | | | | Patients 61-90 years-old | | | |
| --- | --- | --- | --- | --- | --- | --- | --- | --- | --- | --- | --- | --- | --- | --- | --- |
|  |  | **r** | **P** | **OR** | **95% CI** | | **P** | **OR** | **95% CI** | | **P** | **OR** | **95% CI** | | **P** |
| SOLUBLE | p-mol/phen HCL/gua | 0.0410 | 0.359 | 0.7256 | 0.4296 | 1.2259 | 0.2306 | 883523 | 0.8333 | 2.4608 | 0.1937 | 0.9594 | 0.3248 | 2.8340 | 0.9403 |
|  | p-mol/asc ac/phen HCL | 0.109 | 0.0150 | 0.6357 | 0.3751 | 1.0775 | 0.0924 | 1.0970 | 0.6229 | 1.9319 | 0.7485 | 3.0317 | 1.3296 | 6.9124 | 0.0084 |
|  | p-mol/phen HCL/phen mal | -0.0169 | 0.706 | 0.9130 | 0.5261 | 1.5846 | 0.7464 | 1.4016 | 0.8006 | 2.4542 | 0.2375 | 0.2240 | 0.0300 | 1.6729 | 0.1447 |
|  | p-mol/phen HCL | 0.0159 | 0.723 | 0.9051 | 0.5157 | 1.5888 | 0.7285 | 1.0804 | 0.5983 | 1.9511 | 0.7976 | 1.1254 | 0.3778 | 3.3231 | 0.8374 |
|  | p-mol/asc ac/phen mal | -0.0588 | 0.188 | 1.4052 | 0.7344 | 2.6885 | 0.3042 | 0.8688 | 0.4465 | 1.6906 | 0.6789 | 0.2938 | 0.0392 | 2.2025 | 0.2333 |
|  | meta mg | 0.0265 | 0.554 | 0.9218 | 0.4127 | 2.0586 | 0.8425 | 0.8460 | 0.3498 | 2.0462 | 0.7106 | 1.9955 | 0.5663 | 7.0317 | 0.2823 |
|  | acet sal ac/phen HCL | -0.0210 | 0.640 | 1.6519 | 0.4414 | 6.1819 | 0.4560 | 0.4806 | 0.1040 | 2.2206 | 0.3480 | 1.3939 | 0.1741 | 11.1600 | 0.7543 |
|  | others | 0.176 | <0.001 | 0.4079 | 0.1811 | 0.9187 | 0.0304 | 0.7602 | 0.2973 | 1.9436 | 0.5669 | 9.8913 | 3.8390 | 25.4854 | <0.001 |
| OTC anti-common cold products | | **Pearson chi^2^** | | **Patients 18-40 years-old** | | | | **Patients 41-60 years-old** | | | | **Patients 61-90 years-old** | | | |
|  |  | **r** | **P** | **OR** | **95% CI** | | **P** | **OR** | **95% CI** | | **P** | **OR** | **95% CI** | | **P** |
| TABLETS/ CAPSULES | inosine pranobex | -0.0439 | 0.326 | 1.0453 | 0.7137 | 1.5310 | 0.8200 | 1.1836 | 0.7954 | 1.7611 | 0.4059 | 0.4023 | 0.1619 | 0.9997 | 0.0499 |
|  | p-mol/p-ephed/dex HBr | -0.0463 | 0.301 | 1.2385 | 0.7416 | 2.0684 | 0.4136 | 0.9116 | 0.5346 | 1.5543 | 0.7338 | 0.5482 | 0.1626 | 1.8486 | 0.3324 |
|  | ibuprofen/p-ephed | -0.0830 | 0,0633 | 1.9172 | 1.0756 | 3.4173 | 0.0273 | 0.4920 | 0.2609 | 0.9279 | 0.0248 | 0.8780 | 0.2978 | 2.5882 | 0.8135 |
|  | homeopathic products | -0.0439 | 0.326 | 2.2896 | 0.9792 | 5.3537 | 0.0560 | 0.2124 | 0.0640 | 0.7050 | 0.0114 | 2.1027 | 0.6020 | 6.3898 | 0.1900 |
|  | p-mol/phen HCL/asc ac | 0.0985 | 0.0273 | 0.4862 | 0.2290 | 1.0320 | 0.0604 | 1.5300 | 0.7041 | 3.3251 | 0.2829 | 2.6430 | 0.8583 | 8.1385 | 0.0903 |
|  | p-mol/p-ephed/chlorphen | 0.0546 | 0.222 | 0.6958 | 0.2987 | 1.6208 | 0.4006 | 1.0701 | 0.4308 | 2.6582 | 0.8839 | 2.4161 | 0.6771 | 8.6217 | 0.1741 |
|  | acet sal ac | -0.0372 | 0.406 | 1.802 | 0.5787 | 5.6120 | 0.3096 | 0.5125 | 0.1451 | 1.8106 | 0.2992 | 0.9479 | 0.1216 | 7.3915 | 0.9593 |
|  | acet sal ac/asc ac | -0.0736 | 0.0995 | 2.6109 | 0.7401 | 9.2111 | 0.1357 | 0.5125 | 0.1451 | 1.8106 | 0.2992 | 0.4122 | 0.0242 | 7.0156 | 0.5400 |
|  | acet sal ac/p-ephed | 0.00398 | 0.929 | 0.7192 | 0.2455 | 2.1068 | 0.5478 | 1.8643 | 0.6353 | 5.4708 | 0.2568 | 0.5008 | 0.0292 | 8.5914 | 0.6335 |
|  | herbal products | -0.0575 | 0.198 | 2.4921 | 0.5325 | 11.6629 | 0.2462 | 0.5355 | 0.1143 | 2.5091 | 0.4280 | 0.6356 | 0.0366 | 11.0367 | 0.7557 |
|  | p-ephed/tripo | -0.0814 | 0.0683 | 8.3595 | 0.4746 | 147.2368 | 0.1468 | 0.1590 | 0.0090 | 2.8030 | 0.2091 | 0.9831 | 0.0549 | 17.6078 | 0.9907 |
|  | others | 0.127 | 0.00444 | 0.4365 | 0.1847 | 1.0316 | 0.0589 | 1.4448 | 0.4569 | 2.8687 | 0.7729 | 5.1357 | 1.7570 | 15.0119 | 0.0028 |
| OTC anti-common cold products | | **Pearson chi^2^** | | **Patients 18-40 years-old** | | | | **Patients 41-60 years-old** | | | | **Patients 61-90 years-old** | | | |
|  |  | **r** | **P** | **OR** | **95% CI** | | **P** | **OR** | **95% CI** | | **P** | **OR** | **95% CI** | | **P** |
| ADD-ON | xylometazoline HCL | -0.0524 | 0.241 | 1.2126 | 0.8135 | 1.8075 | 0.3438 | 0.9157 | 0.6030 | 1.3906 | 0.6796 | 0.6181 | 0.2605 | 1.4664 | 0.2750 |
|  | oxymetazoline HCL | -0.0117 | 0.794 | 1.0963 | 0.5344 | 2.2490 | 0.8019 | 0.9333 | 0.4381 | 1.9879 | 0.8579 | 0.8864 | 0.2028 | 3.8738 | 0.8727 |
|  | ambroxol HCL | -0.000699 | 0.988 | 1.1055 | 0.6576 | 1.8582 | 0.7052 | 0.8015 | 0.4577 | 1.4034 | 0.4387 | 1.3983 | 0.5534 | 3.5330 | 0.4784 |
|  | bromohexine HCL | -0.00417 | 0.926 | 0.8867 | 0.4442 | 1.7699 | 0.7331 | 1.3521 | 0.6686 | 2.7346 | 0.4012 | 0.4028 | 0.0534 | 3.0399 | 0.3779 |
|  | acetylcysteine | 0.219 | <0.001 | 0.2563 | 0.1015 | 0.6475 | 0.0040 | 0.9743 | 0.3704 | 2.5624 | 0.9579 | 12.2542 | 4,6210 | 32.4962 | <0.001 |
|  | loratadine | -0.0450 | 0.314 | 2.7500 | 0.3188 | 23.7252 | 0.3575 | 0.4841 | 0.0561 | 4.1803 | 0.5096 | 1.1368 | 0.0626 | 20.6396 | 0.9309 |
|  | fexofenadine HCL | -0.00543 | 0.903 | 1.4239 | 0.5830 | 3.4776 | 0.4379 | 0.4494 | 0.1515 | 1.3327 | 0.1492 | 2.1867 | 0.6170 | 7.7497 | 0.2255 |
|  | cetirizine di-HCL | -0.00338 | 0.940 | 1.2767 | 0.3260 | 4.9998 | 0.7258 | 0.6042 | 0.1267 | 2.7898 | 0.5271 | 1.7111 | 0.2098 | 13.9560 | 0.6159 |
|  | antibacterial/antifungal agents | 0.0507 | 0.257 | 0.9023 | 0.5296 | 1.5374 | 0.7054 | 0.8065 | 0.4482 | 1.4513 | 0.4713 | 2.4289 | 1.0387 | 5.6799 | 0.0406 |
|  | choline salicylate | 0.0195 | 0.664 | 0.9311 | 0.3598 | 2.4092 | 0.8830 | 0.8663 | 0.3062 | 2.4504 | 0.7867 | 1.8418 | 0.4058 | 8.3583 | 0.4287 |
|  | others | 0.0627 | 0.161 | 0.8508 | 0.3239 | 2.2350 | 0.7429 | 0.4769 | 0.1360 | 1.6728 | 0.2475 | **4.8360** | **1.4904** | **15.6920** | **0.0087** |

Abbreviations: *acet sal ac* – acetylsalicylic acid; *asc ac* – ascorbic acid; *chlorphen* – chlorpheniramine; *dex HBr* – dextromethorphan hydrobromide; *gua* – guaifenesin; *meta mg* – metamizole magnesium; *p-mol* – paracetamol; *phen HCL* – phenylephrine HCL; *phen mal* - pheniramine maleate; *p-ephed* – pseudoephedrine; *tripro* – triprolidine.
