# Supplementary material for: The impact of patient age on the pharmacist’s recommendation of OTC anti-common cold products before and during the COVID-19 pandemic
Source: Front Pharmacol. 2026 Aug 11;17:1856389. doi: 10.3389/fphar.2026.1856389 (PMC13503212; doi:10.3389/fphar.2026.1856389)
Supplement: Supplementary file 3 [file Table2.docx]

**Supplementary Table S2. Correlation of patient’s age and OTC anti-common cold medications recommendation frequency during COVID-19 pandemic.**

| OTC anti-common cold products | | Pearson chi^2^ | | Patients 18-40 years-old | | | | Patients 41-60 years-old | | | | Patients 61-90 years-old | | | |
| --- | --- | --- | --- | --- | --- | --- | --- | --- | --- | --- | --- | --- | --- | --- | --- |
|  |  | **r** | **P** | **OR** | **95% CI** | | **P** | **OR** | **95% CI** | | **P** | **OR** | **95% CI** | | **P** |
| SOLUBLE | p-mol/phen HCL/gua | -0,0382 | 0,394 | 1.0021 | 0.3672 | 2.742 | 0.9967 | 0.5158 | 0.1639 | 1.6228 | 0.2576 | 5.3538 | 1.4120 | 20.3002 | 0.0136 |
|  | p-mol/asc ac/phen HCL | 0,133 | 0,00283 | 0.3804 | 0.2016 | 0.7176 | 0.0028 | 2.4422 | 1.3175 | 4.5268 | 0.0046 | 1.5140 | 0.4323 | 5.3018 | 0.5166 |
|  | p-mol/phen HCL/phen mal | 0,0341 | 0,447 | 0.8211 | 0.3966 | 1.700 | 0.5956 | 1.1492 | 0.5498 | 2.4019 | 0.7116 | 1.5873 | 0.3536 | 7.1246 | 0.5464 |
|  | p-mol/phen HCL | -0,042 | 0,348 | 1.3674 | 0.6898 | 1.705 | 0.3700 | 0.7101 | 0.3494 | 1.4428 | 0.3439 | 1.1667 | 0.2630 | 5.1750 | 0.8393 |
|  | p-mol/asc ac/phen mal | 0,0343 | 0,444 | 0.8257 | 0.3897 | 1.7494 | 0.6171 | 1.1209 | 0.5232 | 2.4013 | 0.7691 | 1.4713 | 0.3451 | 6.9046 | 0.5701 |
|  | meta mg | 0,0907 | 0,0427 | 0.4227 | 0.1396 | 1.2799 | 0.1277 | 1.5989 | 0.5520 | 4.6312 | 0.3870 | 3.6905 | 0.7759 | 17.5044 | 0.1008 |
|  | acet sal ac/phen HCL | 0,0522 | 0,244 | 0.7745 | 0.2463 | 2.4356 | 0.6621 | 0.7842 | 0.2329 | 2.6403 | 0.6947 | 4.4476 | 0.9162 | 21.5911 | 0.0641 |
|  | others | 0,135 | 0,00255 | 0.4069 | 0.2062 | 0.8031 | 0.0095 | 1.9422 | 1.0067 | 3.7472 | 0.0477 | 2.6586 | 0.8573 | 8.2453 | 0.0904 |
| OTC anti-common cold products | | **Pearson chi^2^** | | **Patients 18-40 years-old** | | | | **Patients 41-60 years-old** | | | | **Patients 61-90 years-old** | | | |
|  |  | **r** | **P** | **OR** | **95% CI** | | **P** | **OR** | **95% CI** | | **P** | **OR** | **95% CI** | | **P** |
| TABLETS/ CAPSULES | inosine pranobex | 0,149 | 0,000836 | 0.3943 | 0.2380 | 0.6532 | 0.0003 | 2.5117 | 1.5280 | 4.1286 | 0.0003 | 1.1842 | 0.3814 | 3.5825 | 0.7647 |
|  | p-mol/p-ephed/dex HBr | -0,0187 | 0,677 | 1.0421 | 0.5206 | 2.0862 | 0.9072 | 1.1983 | 0.5980 | 2.4012 | 0.6099 | 0.2652 | 0.0158 | 4.4582 | 0.3566 |
|  | ibuprofen/p-ephed | -0,098 | 0,0285 | 1.3094 | 0.7613 | 2.2522 | 0.3300 | 0.8267 | 0.4758 | 1.4364 | 0.4996 | 0.6495 | 0.1486 | 2.8393 | 0.5664 |
|  | homeopathic products | 0,13 | 0,00366 | 0.3764 | 0.1389 | 1.0194 | 0.0546 | 1.2731 | 0.4935 | 3.2841 | 0.6175 | 6.9624 | 2.0924 | 23.1669 | 0.0016 |
|  | p-mol/phen HCL/asc ac | 0,0359 | 0,423 | 0.8515 | 0.3549 | 2.0430 | 0.7188 | 0.9694 | 0.3942 | 2.3836 | 0.9460 | 2.2957 | 0.5015 | 10.5101 | 0.2843 |
|  | p-mol/p-ephed/chlorphen | 0,0285 | 0,525 | 0.5961 | 0.2184 | 1.6268 | 0.3125 | 2.0780 | 0.7609 | 5.6747 | 0.1536 | 0.5951 | 0.0346 | 10.2255 | 0.7206 |
|  | acet sal ac | 0,0449 | 0,316 | 0.6257 | 0.2546 | 1.5378 | 0.3067 | 1.6087 | 0.6569 | 3.9396 | 0.2982 | 1.0957 | 0.1402 | 8.5618 | 0.9306 |
|  | acet sal ac/asc ac | 0,00464 | 0,918 | 1.1417 | 0.5187 | 2.5127 | 0.7420 | 0.8270 | 0.3611 | 1.8943 | 0.6533 | 1.7191 | 0.3822 | 7.7580 | 0.4792 |
|  | acet sal ac/p-ephed | -0,018 | 0,689 | 0.7778 | 0.1087 | 5.5661 | 0.8024 | 0.5233 | 0.0530 | 5.0674 | 0.5761 | 7.1818 | 0.7177 | 71.8642 | 0.0934 |
|  | herbal products | 0,107 | 0,0162 | 0.2871 | 0.1007 | 0.8180 | 0.0195 | 2.0245 | 0.7847 | 5.2226 | 0.1447 | 4.6200 | 1.2367 | 17.2595 | 0.0229 |
|  | p-ephed/tripo | -0,0962 | 0,0314 | 3.1795 | 0.6683 | 15.1266 | 0.1461 | 0.3880 | 0.0815 | 1.8467 | 0.2343 | 0.9514 | 0.0541 | 16.7320 | 0.9728 |
|  | others | 0,0918 | 0,0401 | 0.6837 | 0.4577 | 1.0213 | 0.0633 | 1.4465 | 0.9648 | 2.1688 | 0.0740 | 1.5886 | 0.6365 | 3.9647 | 0.3212 |
| OTC anti-common cold products | | **Pearson chi^2^** | | **Patients 18-40 years-old** | | | | **Patients 41-60 years-old** | | | | **Patients 61-90 years-old** | | | |
|  |  | **r** | **P** | **OR** | **95% CI** | | **P** | **OR** | **95% CI** | | **P** | **OR** | **95% CI** | | **P** |
| ADD-ON | xylometazoline HCL | -0,00493 | 0,913 | 1.1387 | 0.7264 | 1.7850 | 0.5711 | 0.8663 | 0.5469 | 1.3723 | 0.5409 | 1.1624 | 0.4206 | 3.2127 | 0.7717 |
|  | oxymetazoline HCL | 0,00322 | 0,943 | 0.7786 | 0.0484 | 12.5186 | 0.8598 | 0.7786 | 0.0484 | 12.5186 | 0.8598 | 4.0468 | 0.1889 | 86.7011 | 0.3713 |
|  | ambroxol HCL | 0,0971 | 0,0299 | 0.4121 | 0.1862 | 0.9118 | 0.0287 | 2.2022 | 1.0184 | 4.7625 | 0.0448 | 1.6520 | 0.3674 | 7.4285 | 0.5128 |
|  | bromohexine HCL | -0,00679 | 0,88 | 0.9905 | 0.6040 | 1.6242 | 0.9698 | 1.061 | 0.6425 | 1.7520 | 0.8171 | 0.8438 | 0.2444 | 2.9132 | 0.7881 |
|  | acetylcysteine | -0,0242 | 0,59 | 1.1766 | 0.4724 | 2.9306 | 0.7269 | 1.0538 | 0.4228 | 2.6265 | 0.9105 | 0.4748 | 0.0279 | 8.0940 | 0.6067 |
|  | loratadine | 0,0367 | 0,413 | 0.7770 | 0.1553 | 3.8878 | 0.7587 | 0.7865 | 0.1427 | 4.3354 | 0.7827 | 4.2909 | 0.4806 | 38.3102 | 0.1922 |
|  | fexofenadine HCL | -0,0292 | 0,514 | 1.3100 | 0.4686 | 3.6617 | 0.6067 | 0.9447 | 0.3378 | 2.6422 | 0.9136 | 0.5951 | 0.0346 | 10.2255 | 0.7205 |
|  | cetirizine di-HCL | 0,0189 | 0,673 | 0.6660 | 0.2206 | 2.0104 | 0.4708 | 1.8717 | 0.6195 | 5.6545 | 0.2665 | 0.7321 | 0.0422 | 12.6937 | 0.8303 |
|  | antibacterial/antifungal agents | 0,141 | 0,00162 | 0.1498 | 0.0325 | 0.6910 | 0.0149 | 3.2473 | 0.9542 | 10.8170 | 0.0595 | 4.4476 | 0.9162 | 21.5911 | 0.0641 |
|  | choline salicylate | 0,0678 | 0,13 | 0.3853 | 0.0699 | 2.1233 | 0.2734 | 1.5864 | 0.3169 | 7.9407 | 0.5744 | 4.2909 | 0.4806 | 38.3102 | 0.1922 |
|  | others | 0,0621 | 0,166 | 0.6266 | 0.4343 | 0.9041 | 0.0124 | 1.3670 | 0.9393 | 1.9894 | 0.1024 | 0.9992 | 0.4151 | 2.4054 | 0.9986 |

Abbreviations: *acet sal ac* – acetylsalicylic acid; *asc ac* – ascorbic acid; *chlorphen* – chlorpheniramine; *dex HBr* – dextromethorphan hydrobromide; *gua* – guaifenesin; *meta mg* – metamizole magnesium; *p-mol* – paracetamol; *phen HCL* – phenylephrine HCL; *phen mal* - pheniramine maleate; *p-ephed* – pseudoephedrine; *tripro* – triprolidine.
